# Peripheral immune cell reactivity and neural response to reward in patients with depression and anhedonia

**DOI:** 10.1038/s41398-021-01668-1

**Published:** 2021-11-05

**Authors:** Sara Costi, Laurel S. Morris, Abigail Collins, Nicolas F. Fernandez, Manishkumar Patel, Hui Xie, Seunghee Kim-Schulze, Emily R. Stern, Katherine A. Collins, Flurin Cathomas, Michael K. Parides, Alexis E. Whitton, Diego A. Pizzagalli, Scott J. Russo, James W. Murrough

**Affiliations:** 1 Depression and Anxiety Center for Discovery and Treatment, Department of Psychiatry, Icahn School of Medicine at Mount Sinai, New York, NY USA; 2 Human Immune Monitoring Center, Icahn School of Medicine at Mount Sinai, New York, NY USA; 3 Tisch Cancer Institute, Icahn School of Medicine at Mount Sinai, New York, NY USA; 4 Department of Psychiatry, New York University School of Medicine, New York, NY USA; 5 Nathan Kline Institute for Psychiatric Research, Orangeburg, NY USA; 6 Department of Neuroscience, Icahn School of Medicine at Mount Sinai, New York, NY USA; 7 Montefiore Medical Center/Albert Einstein College of Medicine, New York, NY USA; 8 Department of Psychiatry, McLean Hospital and Harvard Medical School, Belmont, MA USA; 9 School of Medical Sciences, The University of Sydney, Sydney, NSW Australia; 10 Center for Affective Neuroscience, Department of Neuroscience, Icahn School of Medicine at Mount Sinai, New York, NY USA

**Keywords:** Depression, Physiology, Molecular neuroscience, Human behaviour

## Abstract

Increased levels of peripheral cytokines have been previously associated with depression in preclinical and clinical research. Although the precise nature of peripheral immune dysfunction in depression remains unclear, evidence from animal studies points towards a dysregulated response of peripheral leukocytes as a risk factor for stress susceptibility. This study examined dynamic release of inflammatory blood factors from peripheral blood mononuclear cells (PBMC) in depressed patients and associations with neural and behavioral measures of reward processing. Thirty unmedicated patients meeting criteria for unipolar depressive disorder and 21 healthy control volunteers were enrolled. PBMCs were isolated from whole blood and stimulated ex vivo with lipopolysaccharide (LPS). Olink multiplex assay was used to analyze a large panel of inflammatory proteins. Participants completed functional magnetic resonance imaging with an incentive flanker task to probe neural responses to reward anticipation, as well as clinical measures of anhedonia and pleasure including the Temporal Experience of Pleasure Scale (TEPS) and the Snaith-Hamilton Pleasure Scale (SHAPS). LPS stimulation revealed larger increases in immune factors in depressed compared to healthy subjects using an aggregate immune score (*t*_49 _= 2.83, *p* = 0.007). Higher peripheral immune score was associated with reduced neural responses to reward anticipation within the ventral striatum (VS) (*r* = −0.39, *p* = 0.01), and with reduced anticipation of pleasure as measured with the TEPS anticipatory sub-score (*r* = −0.318, *p* = 0.023). Our study provides new evidence suggesting that dynamic hyper-reactivity of peripheral leukocytes in depressed patients is associated with blunted activation of the brain reward system and lower subjective anticipation of pleasure.

## Introduction

Major depressive disorder (MDD) is a common, chronic and debilitating condition characterized by affective, cognitive, and behavioral symptoms [1, 2]. Despite its large health impact, the neurobiological mechanisms underlying this disorder are still largely unknown. Anhedonia, the reduced ability to experience pleasure, is a core symptom of this disorder and is associated with reduced neural response to reward, poor response to antidepressant medication, and increased risk for suicide [3–5].

Convergent lines of research suggest a dysfunctional immune response in the context of clinical depression [6–9], more specifically linked to anhedonia [10, 11]. Over one-third of patients treated with interferon alpha develop significant depressive symptoms and anhedonia [12, 13] and several studies and meta-analyses have reported an association between depression and increased circulating levels of pro-inflammatory cytokines, most consistently interleukin-6 (IL-6) and tumor necrosis factor alpha (TNF-alpha) [14–16]. Further, there is evidence that childhood adversity, a well-established risk factor for depression [17], is associated with long-term inflammation [18, 19].

Recent work has also connected peripheral immune dysfunction to brain systems that mediate key aspects of depression, such as reward processing [20, 21]. Despite this evidence, the current data appears heterogenous and the precise nature of inflammatory dysregulation in the context of depression remains unclear. Preclinical data show that levels of peripheral cytokines predict the development of anhedonic-like behavior [10]. Repeated exposures to social defeat stress (a well-validated animal model of depression) in rodents causes anhedonia-like behavior characterized by social avoidance and reduced interest in reward in a subset of mice (‘susceptible’ mice); in contrast, ‘resilient’ mice do not develop such behavior. In this model, cytokine profiles 20 min following the first exposure to the stressor (an aggressor mouse) showed that IL-6 was significantly elevated in animals that later exhibited the susceptible phenotype compared with control and resilient mice. Leukocytes collected prior to stress exposure from animals that later developed a susceptible phenotype revealed a significant increase in IL-6 in response to ex vivo stimulation with the bacterial endotoxin lipopolysaccharide (LPS) compared to animals that showed a resilient phenotype [10]. LPS is a major constituent of the membrane of gram-negative bacteria and strongly stimulates innate immunity in diverse species, including humans [22]. LPS, acting as an agonist on Toll-like receptors (TLR)-4 localized on the surface of myeloid cells, induces a dynamic response leading to the production of inflammatory cytokines and chemokines. While previous studies have shown that LPS administration is capable of inducing depressive-like symptoms [23] in healthy subjects, the specific question of leukocyte cell reactivity to an immune challenge in depressed subjects has been understudied. Interestingly, in vitro lymphocytic activity has been previously explored in the context of depression with depressed subjects showing a decrease in vitro lymphocyte activity [24].

Building on these data, the current study aimed to translate preclinical findings into humans by investigating a large panel of peripheral immune factors in subjects with depression from peripheral blood mononuclear cells (PBMC) stimulated ex vivo with LPS. To test specific immune-behavior relationships with anhedonia, subjects with unipolar depression and healthy volunteers completed a multilevel assessment of anhedonia using rating scales, a computer-based task [probabilistic reward task (PRT) [25, 26]], and a task-based functional magnetic resonance imaging (fMRI) measure of reward anticipation (incentive flanker task, IFT) [27–29], with a particular focus on the ventral striatum (VS). We examined if LPS stimulation revealed larger increases in immune factors in depressed subjects compared to healthy volunteers. We further explored whether LPS-stimulated immune factors were associated with objective and self-reported measures of anhedonia and neural reactivity to reward anticipation.

## Materials and methods

### Study participants and design

The study was conducted at the Icahn School of Medicine at Mount Sinai (ISMMS) in New York City wherein participants were recruited between September 2016 and August 2018. The institutional review board at ISMMS approved the study, and written informed consent was obtained prior to any study procedure. Participants were between the ages of 18 and 55 and met DSM-5 criteria for a primary diagnosis of major depressive disorder (MDD), persistent depressive disorder (PDD), or other specified depressive disorder, as assessed by the Structured Clinical Interview for DSM-5 Axis I Disorders – Patient Edition (SCID-I/P) [30]. A healthy control (HC) group with similar age, sex, and body mass index (BMI) was also enrolled, as these variables are well-known to affect inflammatory markers [31–37]. Exclusionary criteria included a history of inflammatory or autoimmune disorder, clinically significant abnormalities of laboratories, and any unstable medical condition. Treatment with psychotropic medication or systemic steroids within 4 weeks of assessment, or use of medication or nutritional supplement known to affect the immune system within one week of the assessment visit, were exclusionary. A complete list of the inclusion and exclusion criteria is available in Supplemental Material.

During the screening visit, medical and psychiatric history was obtained. All participants underwent clinical laboratory tests and toxicology screening. A pregnancy test was performed in premenopausal women. Within 4 weeks of screening, all eligible participants completed the assessment visit, which included a blood draw for inflammatory markers collection, and clinical and neuro-behavioral assessment of depression and anhedonia.

### Clinical assessment of anhedonia

Anhedonia was assessed using the Snaith-Hamilton Pleasure Scale (SHAPS) [38], a well-validated self-report questionnaire assessing pleasure capacity, both consummatory and anticipatory. Hedonic capacity was also measured using the Temporal Experience of Pleasure Scale (TEPS) [39], a validated self-report that provides specific sub-scores of anticipatory and consummatory pleasure. A higher score of the SHAPS indicates more severe anhedonia; a higher score on the TEPS indicates greater pleasure and hence lower anhedonia severity.

### Blood collection and processing

Blood was collected between 8 AM and 10 AM into tubes containing ACD Solution A as anti-coagulant (BD Vacutainer) and delivered to the Human Immune Monitoring Center (HIMC) of ISMMS for processing and storage. To mitigate the effect of confounding factors, participants were asked to reduce the intake of lipids and to avoid alcoholic beverages in the 12 and 24 h, respectively, prior to the blood draw. Details on the blood processing procedures are available in Supplemental Material.

The samples were analyzed mid-way through the study and at the end of the study period using Olink multiplex assay – Inflammatory panel (Olink Bioscience, Uppsala, Sweden), according to the manufacturer’s instructions. The inflammatory panel allows the detection of 92 proteins associated with human inflammatory conditions. More details are included in the Supplemental Material.

### MRI acquisition, processing, and reward task (incentive flanker task)

All MRI data were acquired with a Siemens 3T MAGNETOM Skyra scanner and a 32-channel head coil at ISMMS. Functional scans were preprocessed and denoised for motion and physiological noise using multi-echo independent component analysis (ME-ICA) [40, 41]. Multi-echo functional MRI data were decomposed into independent components, and scaled against TE [40–42]. Components with high TE-dependence are considered BOLD-like whereas components with low TE-dependence are considered noise-like [40–42]. Removal of non-BOLD components allows robust data denoising for motion, physiological, and scanner artifacts [42]. The incentive flanker task (IFT) is a modification of the monetary incentive delay (MID) task [29]. Further details, including a detailed description of the task, are included in the Supplemental Material (Fig. S1).

### Probabilistic reward task

The probabilistic reward task (PRT) [25] is a signal detection test that provides an objective assessment of reward learning and was completed on the assessment visit. A detailed description of the task is provided in the Supplemental Material.

### Statistical analyses

The sample size was calculated using anhedonia as a continuous outcome and relying on preliminary data wherein the magnitude of association between a linear combination of immune factors provided >90% power to detect an association between immune factors and anhedonia based on a proposed sample size of *n* = 50 (MDD, *n* = 30; HC, *n* = 20).

Demographic and clinical characteristics, blood biomarkers, and behavioral measures of reward are summarized separately. Continuous variables are summarized using means and standard deviations, while categorical variables are summarized as proportions. Cytokines and chemokines with >50% missing or below the limit of detection (LOD) values were excluded from the analysis. Outliers, defined as standard deviations >3, were excluded separately for each immune factor [43]. Each of the remaining proteins had <5% missing data, considered randomly missing. Predictive mean matching was used for imputation of missing data using multivariate imputation by chained equations (MICE) using R software [44], which computes a realistic distribution, separately optimized for each variable, and randomly selects a regression-predicted value from 50 iterations [45–47]. A list of analytes that were not included in the analysis is available in the Supplemental Material. To determine the influence of stimulated levels of immune factors, the proteins’ fold change was calculated as the value of the cytokine released by PBMC following LPS stimulation divided by the un-stimulated value of the cytokines secreted by PBMC prior to the LPS challenge. The resultant fold-change protein data (42 proteins × 51 subjects) were entered into principal component analysis (PCA) to reduce the dimensionality of the multidimensional data, while retaining the majority of variability within the data. A scree plot of eigenvalues was computed and inspected to indicate component significance (see Supplemental Material). Significant components from the PCA were examined for group differences based on individual factor loadings using independent-samples *t*-test, and used for correlations with self-report questionnaires, clinician-administrated scales, measures of reward learning, and brain activation using two-tailed Pearson’s correlation. The PCA was used for the statistical analysis and results.

Task-based fMRI analysis included first-level general linear models (GLM) with regressors for cue onset (reward/loss/neutral), flanker onset, and feedback onset, each including duration modulation using AFNI’s stim_DM function, and convolved with the hemodynamic response function. The contrast of interest for reward anticipation was gain cue > neutral cue. Activation for each contrast was extracted within the VS ROI from FSL’s Harvard-Oxford atlas [48] for each subject and entered into independent samples *t*-test for group difference comparison. Pearson correlation was used to examine relationships between VS activation during reward anticipation and significant immune principal components. An additional whole-brain analysis was performed using two-tailed Pearson’s correlation between whole-brain activation maps for gain > neutral cue and the first significant immune principal component (PC1) factor loading. For this, a voxelwise, cluster-defining threshold of *p* < 0.005 was used plus cluster-wise correction of K > 60 as calculated by AFNI’s ACF function to mitigate against spatial autocorrelation and the incidence of false-positives [49].

## Results

### Sample characteristics

Of the 57 subjects assessed for eligibility, 51 met inclusion/exclusion criteria and completed the assessment visit: 30 subjects with depression and 21 matching healthy volunteers. Within the depressed group, 28 subjects met criteria for current MDD, one for PDD, and one for other specified depressive disorder. Of these, 28 were in a current major depressive episode (MDE). No significant difference emerged between the two groups in sociodemographic characteristics, age, gender, BMI, or smoking status (Table 1).

**Table 1:** Demographic and clinic characteristic of study sample.

	MDD (*N* = 30)	Healthy controls (*N* = 21)	*p* value
Age, *M* (SD)	37.1 (10.8)	37.1 (9.6)	0.98
Gender (male), *n* (%)	14 (46.7)	13 (61.9)	0.28
BMI, *M* (SD)	26.7 (5.8)	24.8 (4.7)	0.22
Daily smoking, *n* (%)	0 (0)	0 (0)	1
hs-CRP level, *M* (SD)	3.8 (7.2)	1.7 (1.6)	0.19
Race, *n* (%)			
White/Caucasian	16 (53.3)	10 (47.6)	0.08
Ethnicity, *n* (%)			
Hispanic/Latino	7 (23.3)	3 (14.3)	0.22
Employment, *n* (%)			
Employed	21 (70.0)	17 (81.0)	0.05
Education, *n* (%)			
Bachelor Degree	21 (70.0)	16 (76.2)	0.61
Relationship status, *n* (%)			
Married	2 (6.7)	4 (19.1)	0.07
Psychiatric comorbidities			
Anxiety disorder, *n* (%)	14 (46.7)	0 (0)	<0.001
PTSD current, *n* (%)	1 (3.3)	0 (0)	0.22
MADRS, *M* (SD)	27.4 (6.4)	1 (1.6)	<0.001
SHAPS, *M* (SD)	34.5 (7.6)	17.6 (4.9)	<0.001
TEPS anticipatory, *M* (SD)	26.7 (10.5)	45.1 (6.5)	<0.001
TEPS consummatory, *M* (SD)	26.1 (10.5)	39.3 (6.1)	<0.001

Race and ethnicity were reported by the study participants.

*BMI* body mass index, *hs-CRP* high-sensitivity C reactive protein, *M* means, *MADRS* Montgomery–Åsberg Depression Rating Scale, *MDD* Major Depressive Disorder, *PTSD* posttraumatic stress disorder, *SD* standard deviation, *SHAPS* Snaith-Hamilton Pleasure Scale, *TEPS* Temporal Experience of Pleasure Scale.

### Immune markers

Basal plasma factors: Analysis of peripheral immune parameters were conducted utilizing 69 analytes from plasma. There were no group differences in circulating plasma levels of inflammatory factors (see Supplemental Material; Table S1).

**Fig. 1:**
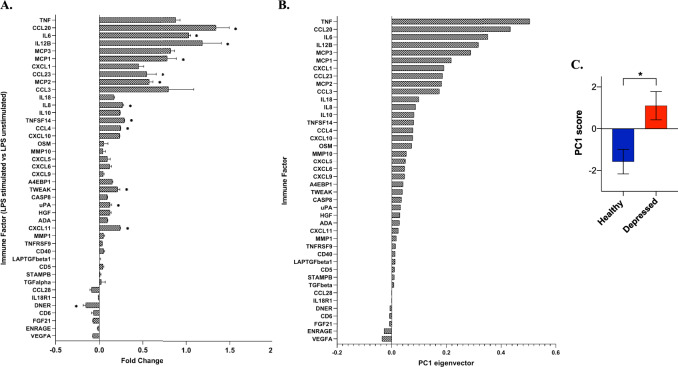
Heightened peripheral blood mononuclear cells reactivity in depression. **A** Difference in fold change of levels of lipopolysaccharide (LPS) stimulated immune factors released ex vivo by peripheral blood mononuclear cells (PBMCs) between depressed and healthy volunteers - uncorrected *t*-test. **B** Principal component 1 (PC1) factor loading. PC1 explained a large proportion of the variance, with an eigenvector of 12.6, accounting for 52.8% of the total variance in the data and included highest factor loadings for tumor necrosis factor alpha (TNF-alpha), chemokine ligand 20 (CCL-20), interleukin-6 (IL-6), interleukin-12 subunit beta (IL-12B), and monocyte-chemotactic protein 3 (MCP3). **C** Immune reactivity PC1 score difference between depressed subjects and healthy volunteers; *p < 0.05, error bars represent standard error of the mean (SEM).

LPS simulated PBMCs: Analysis of peripheral immune parameters was conducted utilizing 42 analytes from supernatant from LPS-stimulated PBMCs, based on the LOD described above. In contrast to the findings with basal plasma levels, patients with depression showed a greater increase in PBMC release of inflammatory factors following stimulation with LPS compared to healthy controls as calculated by the immune markers’ fold change. Immune factors fold change in depressed and healthy controls are reported in Fig. 1A.

LPS-stimulated PBMC analytes were subjected to principal component analysis (PCA). The first three components preceded the scree plot eigenvector elbow, all had an eigenvector value >1 and were thus considered significant. Principle component 1 (PC1) explained a large proportion of the variance (52.8%), with an eigenvector of 12.6 (Fig. S2). PC1 included highest factor loadings for TNF, C-C motif chemokine 20 (CCL20), IL6, interleukin-12 receptor subunit beta (IL12B), and monocyte-chemotactic protein 3 (MCP3) (Fig. 1B). This component was significantly higher in patients with depression compared to controls (*t*_49_ = 2.83, *p* = 0.007, Fig. 1C). The other two significant components, PC2 and PC3 had highest loadings for TNF and IL-12b, respectively, and were not different between groups (*p*’s > 0.3).

### Correlation between immune factors and clinical symptoms

**Fig. 2:**
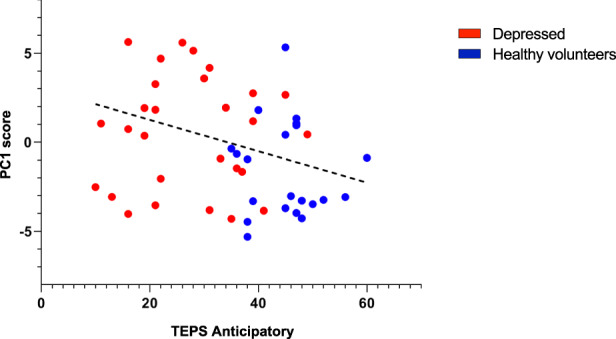
Peripheral blood mononuclear cells reactivity is negatively associated with response to pleasure. Negative correlation between immune factors released by peripheral blood mononuclear cells (PBMCs) stimulated ex vivo with lipopolysaccharide (LPS) represented by factor loading on PC1 and anticipatory reward, as measured with the anticipation subscale of the TEPS self-report.

The relationship between peripheral immune factors and anhedonia was examined across the full sample, and within the two groups separately. For these correlations, BMI, age, and sex were included as covariates. A greater PC1 score was associated with greater anticipatory anhedonia as measured by the TEPS anticipatory sub-score (*r* = −0.34, *p* = 0.016) (Fig. 2). PC1 was not correlated with the TEPS consummatory anhedonia as measured by the TEPS consummatory sub-score (*r* = −0.12, *p* = 0.170) or with general anhedonia as measured by the SHAPS (*r* = 0.25, *p* = 0.071).

### Task-based MRI results

**Fig. 3:**
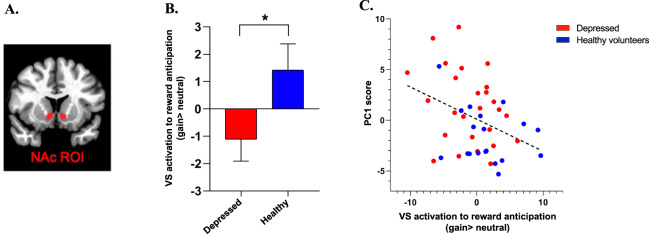
Blunted response to reward in depression and association with immune reactivity. **A** Ventral striatum (VS) region of interest (ROI); **B** Group difference in VS response to reward (gain > neutral cue) during the first run of the task in MDD and HC in VS ROI. **C** Correlation between immune reactivity, as computed through the principal component 1 (PC1) factor loading, and VS activation to reward (gain cue > neutral cue) on the first run of the (incentive flanker task) IFT across the sample.

Functional MRI data during the IFT were available for 28 depressed subjects (age = 36.5 ± 11, 14 females) and 20 HC subjects (age = 37.8 ± 9.4, 7 females). One MDD subject was excluded due to excessive motion during MRI. Groups did not differ in their performance accuracy (MDD = 85.9% ± 10.4, HC = 87.7% ± 0.1, *p* = 0.76), or their baseline reaction times (MDD = 927.6 ms ± 172.5, HC = 859.3 ms ± 145.7, *p* = 0.16). Patients with MDD showed lower VS response to reward (gain cue > neutral cue) compared to controls across all four runs of the task (*t*_45_ = −1.88, *p* = 0.066) but most notably during the first run (*t*_45_ = −2.05, *p* = 0.047), consistent with previous reports of VS habituation to reward over time [50] (Fig. 3). Therefore, follow-up analyses were performed utilizing the first run only.

Consistent with our hypothesis, a higher PC1 immune score was associated with reduced activation to reward anticipation in the VS across both groups (*r* = −0.376, *p* = 0.011; Fig. 3). This association remained significant when controlling for age, gender, and BMI (*r* = −0.389, *p* = 0.011). No correlation emerged within the MDD (*r* = −0.26, *p* = 0.230) or HC group (*r* = −0.3, *p* = 0.250) alone. An additional exploratory whole-brain analysis correlating PC1 against whole-brain activation to gain > neutral cue revealed no significant correlation between brain activation and PC1.

### Behavioral measures of reward (PRT)

Fifty-one subjects completed the PRT on the assessment day. Of these, 17 healthy volunteers and 23 depressed subjects had valid data following the quality check (QC). Reasons for exclusion were: too many outliers (*n* = 8), uncompleted task (*n* = 1), and below chance accuracy (*n* = 2). The remaining analysis was conducted only on the subjects who passed the QC (*n* = 40).

As hypothesized and consistent with prior reports, response bias (computed by subtracting the total response bias in the first block from the total response bias in the last block) was higher in healthy compared to depressed [*t*_38_ = 2.27 *p* = 0.029); Fig. S3]. Reward learning was associated with greater activation to reward anticipation in the VS (*r* = 0.32, *p* = 0.053), but did not correlate with PC1 immune score upon controlling for BMI, age, and sex (*r* = −0.16, *p* = 0.33). For more details, see Supplemental Material.

## Discussion

The current study investigated the relationship between inflammatory factors released by PBMCs stimulated ex vivo with LPS, anhedonia and neural reactivity to reward anticipation in depression. We found that depressed subjects showed an exaggerated response to the immune challenge with LPS compared to HC. Further, an increase in immune factors released by PBMCs following LPS stimulation was associated with reduced anticipation of pleasure as measured with the anticipatory sub-score of the TEPS and with a reduced response to reward anticipation within the VS in the brain.

Depressed subjects showed increased levels of immune markers released by PBMCs stimulated ex vivo with LPS, consistent with previous work showing similar results in rodent stress models of depression [10]. Here, we were able to translate these findings into humans and expand it through the analysis of a broad range of immune factors. Of note, other authors have investigated the effect of LPS stimulation within depressed subjects with mixed results. A large study [51] using data from the Netherlands Study of Depression and Anxiety (NESDA) on adults with current or remitted depression or anxiety disorders and healthy controls explored the correlations between clinical symptoms of anxiety and depression and inflammatory markers that were assayed from plasma and after whole blood stimulation with LPS. In this large cohort, LPS-stimulated inflammatory markers, but not plasma factors, were associated with anxiety symptom, but not with depression severity, upon adjustment for health condition and lifestyle. A recent study [52] on adolescents with anhedonia across a broad spectrum of psychiatric disorders showed no group differences in cytokine levels following whole blood LPS stimulation. Of note, the different LPS stimulation approach (whole blood compared to isolated PBMC stimulation) may explain the different results. Further, we enrolled only adult subjects diagnosed with unipolar depressive disorders to minimize heterogeneity [11, 53]. Others [54] analyzed the levels of pro-inflammatory cytokines IL-6, TNF-alpha, and IL-1beta following LPS stimulation on isolated PBMC on depressed and healthy volunteers reporting a reduced response to LPS stimulation in depressed. The authors isolated CD14 + monocytes prior to culture cultured them with LPS and used a chemiluminescence method to measure the levels of the pro-inflammatory cytokines IL-6, TNF-alpha, and IL-1beta. Different LPS stimulation protocol and analysis methods may account for the dissimilar findings.

We did not find a difference between groups in un-stimulated plasma level in the current study. This negative result is discordant with several studies and meta-analyses showing higher levels of some immune factor in patients with depression compared to non-depressed controls, such as C-reactive protein (CRP), IL-6, and TNF-alpha [14, 15, 55]. Notwithstanding a number positive reports, there have been several negative reports as well, including the large NESDA study discussed above. Indeed meta-analyses suggest that findings of elevated circulation inflammatory factors in patients with depression may be small and quite variable [55, 56]. Recent data suggest that inflammation may be prominent only in a subset of depressed patients and mostly among subjects with treatment resistant depression [57]. A recent study by Syed et al. suggests that lifetime treatment with antidepressant may affect cytokines concentration in MDD [58]. In this work, drug-naive depressed subjects showed an elevation in plasma concentration of pro- and anti-inflammatory cytokines compared to healthy volunteers that was immunosuppressive on freshly isolated PBMCs from a healthy volunteer donor at the monocytes/dendritic cells, B cells and T cell memory level. Further, a 12-week period treatment with escitalopram, duloxetine or cognitive behavioral therapy (CBT) was associated with an increase in anti-inflammatory cytokine levels in both responder and non-responders, whereas pro-inflammatory cytokines continued to increase in non-responders while they were stabilized in responders.

This study is among the firsts to use Olink inflammatory panel to explore a wide range of inflammatory markers within a psychiatric population [59]. The use of this assay, one of the most extensive panel available for proteins associated with inflammation and related biological processes, highlighted the potential contribution of a broad range of immune and inflammatory factors to depression and anhedonia, beyond well-studied pro-inflammatory cytokines such as TNF-alpha and IL-6. Interestingly, there has been increasing interest regarding the role of chemokines in the neurobiology of depression [60]. Chemokines act as chemiotactic factors and activators of peripheral immune cells that can potentially contribute to the neurodegeneration observed in depression [61, 62]. For instance, monocyte-chemotactic proteins (MCP)-1, together with MCP-2 and MCP-3, is well known to exert potent pro-inflammatory action and has been shown to possess the ability to translocate across the blood–brain barrier (BBB) and induce chemotaxis of circulating leukocytes [63]. Thus, broader spectrum analysis of immune markers should be explored to investigate further the biological mechanisms of immune dysregulation in depression.

The second main finding of our study was that the degree of immune cell reactivity was positively associated with impairment in the ability to experience pleasure across groups as measured by self-report. Consistent with the work from Freed et al. [52], the magnitude of immune response was negatively associated with anhedonia severity. Similarly, others investigated the relationship between immune factors released by whole blood LPS stimulated and symptom severity [64] across a spectrum of depressive disorders and showed an association between LPS-stimulated inflammation and depression severity.

Our third finding was that the magnitude of immune cell reactivity was negatively associated with VS activation to reward anticipation. There is substantial evidence that peripheral cytokines impact reward processing areas implicated in anhedonia and depression [65–68]. The administration of pro-inflammatory cytokines (e.g., INF-α) or endotoxin have been shown to reduce neural responses to reward [67, 68], while the relationship between plasma high-sensitivity CRP (hs-CRP) and the brain reward circuit has been investigated in depression, at the level of resting-state functional connectivity [21, 65]. Herein, we showed that greater immune activity was associated with reduced activation to reward anticipation in the VS. These findings suggest immune reactivity in depression may be associated with a specific aspect of the hedonic experience that relies on the brain reward system centered on the VS [39, 69]. However, caution should be taken in generalizing these results given the limited sample size of this study and potential limitations of test-retest reliability of task-based fMRI [70]. Further work focusing on the interaction between the peripheral immune system and the brain reward circuit in larger samples are warranted to improve our understanding on the effect of immune dysregulation on the brain circuit of depressed subjects with anhedonia.

Finally, in the current study, depressed subjects were characterized by a blunted reward learning toward rewards as measured by the PRT, although this was not associated with LPS-stimulated immune factors. This is the first report investigating the relationship between reward learning (as measured with a computer-based task) and cytokine change following PBMCs stimulation with LPS. A recent study exploring the effect of the influenza vaccination on reward learning in 41 young healthy subjects showed that increases in IL-6 in circulation predicted increased performance on the PRT [71]. The different study population and the mild systemic inflammatory response elicited by the influenza vaccine may explain the differences in behavioral sensitivity to inflammation in this sample compared to healthy volunteers.

Limitations of the study include the relatively small sample size and the enrollment of subjects with a broader unipolar depressive phenotype. The small sample size may have led to Type II errors that could explain, for example, the lack of association between LPS-stimulated immune factor and activation to reward anticipation in the depressed group alone. Hence, this data may guide sample size calculation for future larger studies studying dynamic changes in the immune factors and response to reward within depression. Moreover, this study did not explore the effect of antidepressant medication on immune challenged PBMCs. Although limiting the external validity of the study, given the size of the sample analyzed, we opted to limit enrollment to subjects free of medication due to the unknown interactions between CNS medications on immune factors released by PBMC following LPS stimulation and the well-established effects on antidepressants on fMRI measures in depression. Further, since this study focused on currently depressed subjects and lacks a depressed control group in remission, it is unclear if the altered immune profile observed should be considered a state or a trait characteristic of depression. Finally, the current study focused on the isolation of PBMC prior to LPS stimulation, but did not explore the contribution of cellular immune markers. The increased levels of immune markers released by PBMCs stimulated ex vivo with LPS in depressed compared to healthy controls warrants further exploration and suggest a prominent role for the monocyte/macrophage lineage in the context of depression. Future studies investigating the cellular mechanisms associated with depression using a transcriptome approach to examine the molecular mechanisms of peripheral inflammation relevant to depression are needed.

In the current study, we found that changes in immune factors in response to LPS stimulation were associated with depression and correlated with impaired anticipatory reward function at the clinical and neuronal level. Future studies with larger sample sizes are required to improve the knowledge of the neurobiological mechanism underlying depression.

## Supplementary information

Supplemental Information
