# Supplementary material for: Peripheral immune cell reactivity and neural response to reward in patients with depression and anhedonia
Source: Transl Psychiatry. 2021 Nov 5;11:565. doi: 10.1038/s41398-021-01668-1 (PMC8571388; doi:10.1038/s41398-021-01668-1)
Supplement: Supplementary file 1 — Supplemental Information [file 41398_2021_1668_MOESM1_ESM.docx]

**Peripheral immune cell reactivity and neural response to reward in patients with depression and anhedonia**

**Supplemental Information**

**Table of Contents**

**1. Supplemental Methods**

**2. Supplemental Results**

**3. Supplemental Figures**

**4. Supplementary References**

**1. Supplemental Methods**

**Inclusion and Exclusion Criteria**

Participants were between the ages of 18 and 55 and meet DSM-V criteria for major depressive disorder (MDD), persistent depressive disorder (PDD) or other specified depressive disorder, as assessed by a trained rater using the Structured Clinical Interview for Diagnostic and Statistical Manual of Mental Disorders-Fifth Edition Text Revision (DSM-V-TR) Axis I Disorders – Patient Edition (SCID-I/P). If MDD and PDD in a current major depressive episode (MDE) were met concurrently, MDD was indicated as primary diagnosis. A group of non-depressed healthy control volunteers (HC, n=20) was also enrolled. Exclusionary diagnoses included substance use disorder in the past two years, lifetime history of schizophrenia or other psychotic disorder, bipolar disorder, neurodevelopmental disorder or neurocognitive disorder. Lifetime history of inflammatory or autoimmune disorder, including but not limited to rheumatoid arthritis, ankylosing spondylitis, myositis, vasculitis, systemic lupus erythematosus, Sjogren's Syndrome, or scleroderma was also exclusionary. Other exclusion criteria included pregnancy, urine toxicology positive for illicit drugs, smoking habit ≥ 1 package a day, and moderate alcohol use (defined by ≥ 4 drinks at least 3 times a week). Treatment with antidepressant or other psychotropic medication within 4 weeks of assessment visit (8 weeks for fluoxetine), or systemic steroids within 4 weeks of assessment, or use of medication or nutritional supplement known to affect inflammation, including but not limited to non-steroidal anti-inflammatory agents (NSAIDs), aspirin, acetaminophen, COX-2 selective inhibitors, omega-3 fatty acids, turmeric extract, ginger extract, vitamin E, “Devil’s claw” curcumin, or grape derived polyphenols within one week of the assessment visit (V1) were exclusionary. Subjects with clinically significant abnormalities of laboratories (that included complete blood count with differential, glucose level, electrolyte and fluid balance, kidney and liver function, and urinalysis) and any unstable medical illnesses including hepatic, renal, gastroenterologic, respiratory, cardiovascular, endocrinologic, neurologic, immunologic, or hematologic disease were also excluded.

**Blood Processing and Analysis**

Blood was processed within 2 hours of collection and spun at 1500 rpm for 10 minutes. Plasma was collected and the cell fraction was diluted with Phosphate Buffered Saline (PBS) (ratio 1:2). Diluted blood suspension was overlaid over the top of Ficoll-Paque Plus (ratio 7:3) (GE Healthcare Life Sciences) and centrifuged for 20 minutes at 1800 rpm at room temperature. After centrifugation the peripheral blood mononuclear cells (PBMCs) layer was collected, washed in PBS according to the standard operating procedure (SOP) for PBMCs isolation. Plasma was stored at -80º C and PBMCs in Liquid N2 tank until immune based assay commencement. For the LPS Stimulation purified PBMCs were treated with LPS at 0.1 microg/ml in RPMI+5% FBS at 0.5Million cell/well in 96 well tissue culture plate at 37ºC for six hours. Plate was centrifuged to collect the supernatants post six hours incubation. Supernatant was then stored at -80ºC. The samples were analyzed using Olink multiplex assay – Inflammatory panel (Olink Bioscience, Uppsala, Sweden), according to the manufacturer’s instructions. PBMC viability post cryopreservation ranges from 90.5 - 96. 8%

Olink multiplex assay – Inflammatory panel

The inflammatory panel includes 92 proteins associated with human inflammatory conditions. Briefly, an incubation master mix containing pairs of oligonucleotide-labeled antibodies to each protein, was added to the samples and incubated for 16 hours at 4 ºC. Each protein was targeted with two different epitope-specific antibodies increasing the specificity of the assay. Presence of the target protein in the sample brought the partner probes in close proximity, allowing the formation of a double strand oligonucleotide polymerase chain reaction (PCR) target. On the following day, the extension master mix in the sample initiated the specific target sequences to be detected and generated amplicons using PCR in 96 well plate. For the detection of the specific protein, Dynamic array integrated fluidic Circuit (IFC) 96x96 chip was primed, loaded with 92 protein specific primers and mixed with sample amplicons including three inter-plate controls (IPS) and three negative controls (NC). Real time microfluidic qPCR was performed in Biomark (Fluidigm, San Francisco, CA) for target protein quantification. Data analysis was performed by employing a pre- processing normalization procedure using Real time PCR analysis software via ΔΔCt method and Normalized Protein Expression (NPX) manager. For each sample and data point, the corresponding Cq-value for the Extension control was subtracted, thus normalizing for technical variation within one run. Normalization between runs is then performed for each assay by subtracting the corresponding dCq-value for the Interplate Control (IPC) from the dCq-values generated. In the final step of the pre-processing procedure the values are set relative to a correction factor determined by Olink. The generated Normalized Protein eXpression (NPX) unit is on a log2 scale where a larger number represents a higher protein level in the sample, typically with the background level at around zero. One NPX difference equals to the doubling of the protein concentration.

Olink performance characteristic and quality controls

Internal and external controls have been developed by Olink for data normalization and quality control purposes. These controls have been designed to enable monitoring of the technical assay performance, as well as the quality of individual samples, providing information at each step of the Olink protocol. The internal controls are added to each sample and include two Immunoassay controls, one Extension control, and one Detection control. The Immunoassay controls (two non-human proteins) monitor all three steps starting with the immunoreaction. The Extension Control (an antibody linked to two matched oligonucleotides for immediate proximity independent f antigen binding) monitors the extension and readout steps and is used for data normalization across samples. Finally, the Detection control (a synthetic double-stranded template) monitors the readout step. Samples for which one or more of the internal control values deviate from a pre-determined range will be flagged and may be removed before statistical analysis.

Triplicate external controls and inter-plate controls (IPC) are included on each plate and used in a second normalization step. This control is made up of a pool of proves similar to the Extension control (Ext Ctrl), but generated with 92 matching oligonucleotide pairs. Furthermore, this improves inter-assay precision and allows for optimal comparison of data derived from multiple runs. In addition, we included two additional biological reference controls on each plate to facilitate tracking of inter-plate reproducibility and normalize for any potential inter-plate batch effects, as well as recombinant cytokines at different concentration to assess specificity and sensitivity.

**MRI Acquisition and Processing**

All MRI data were acquired with a Siemens 3T MAGNETOM Skyra scanner and a 32-channel head coil at ISSMS’s Translational and Molecular Imaging Institute (TMII). Scans included an anatomical T1-weighted scan, a functional scan during resting state (10min, eyes open) and a task-based functional scan with the Incentive Flanker Task (IFT, detailed below). The anatomical T1-weighted images were acquired with a magnetization-prepared 2 rapid gradient echo (MP2RAGE) sequence, which collects 2 volumes after each inversion for improved image quality (TR=4000ms, TE=1.9, inversion 1/2 time=633/1860, FOV=186x162, voxel resolution= 1x1x1mm). Functional scans were collected with a multi-echo multiband accelerated echo-planar imaging (EPI) sequence (TR=882ms, TE’s=11.0, 29.7, 48.4, 67.1, multi-band factor=5, FOV=560x560, voxel resolution= 3x3x3mm, flip angle=45). Functional scans were preprocessed and denoised for motion and physiological noise using multi-echo independent component analysis (ME-ICA) ^1,2^. Multi-echo functional MRI data were decomposed into independent components, and scaled against TE ^1–3^. Components with high TE-dependence are considered BOLD-like whereas components with low TE-dependence are considered noise-like ^1–3^. Removal of non-BOLD components allows robust data denoising for motion, physiological and scanner artifacts ^3^.

**Incentive flanker task**

The incentive flanker task (IFT) is a modification of the monetary incentive delay (MID) task and described in detail elsewhere ^4^. There were three trial types (reward/loss/neutral). Each trial contains an initial monetary cue (reward/loss/neutral) (2-6s). This was followed by a flanker task, which consisted of a display of five letters in row. Subjects were instructed to respond to the middle letter only by pressing a left button if the middle letter is S or K, and the right button if the middle letter is H or C. Flanker letters could be congruent or incongruent (50/50%) with the middle letter. The response period was titrated based on baseline performance (the mean baseline performance was multiplied by 1.5, with 1700ms maximum). After a button response, feedback was displayed for 2s. Across all trial types, feedback following a correct response was a happy face with the word: ‘Correct!’, whereas feedback following an incorrect response was a sad face with: ‘Incorrect!’. In addition, feedback included monetary value (correct response on reward/loss/neutral = $0.50/$0.00/$0.00; incorrect response on reward/loss/neutral = $0.00/-$0.50/$0.00), and at the end of the session, participants were rewarded with real money based on their performance. One-third of cues were followed by a blank screen for 2s to disrupt collinearity between the cue and feedback. Outcome feedback was followed by a blank inter-trial interval (ITI) for 2–6s before the next trial. There were 40 of each trial type (reward/loss/neutral), producing a total of 120 trials. Trials were presented in pseudorandom order and equally divided across 4 runs of approximately 6 min each. A schematic representation of task is included in Figure S1 of the Supplementary Material.

**Probabilistic Reward Task**

The Probabilistic Reward Task (PRT) ^5^ is a signal detection test that provides an objective assessment of reward learning and was completed by all study participants on the assessment visit. The task consisted of three 100-trial blocks and was completed on a 17” PC monitor using E-Prime (version 1.1; Psychology Software Tools, Inc, Pittsburgh, PA). Participants were presented with simple cartoon faces with either a straight nose or mouth of 11.5 mm (“short”) or 13 mm (“long”) for 100 ms and instructed to press an appropriate button to decide whether a long or small mouth had been presented in order to receive a monetary reward of 20¢. Unbeknownst to subjects, correct identification of one stimulus (the “rich stimulus”) was rewarded three times more frequently (“Correct! You won 20 cents”) than the other (“lean”) stimulus. The degree of response bias toward the more frequently reinforced stimulus was used for operationalizing response to reward. Healthy subjects reliably develop a response bias for the rich stimulus, regardless of which stimulus is actually presented. Subjects with MDD, however, tend to respond similarly to both stimuli, and fail to develop this bias for the more frequently reinforced stimulus, thus indicating decreased responsiveness to rewards ^6,7^. An index of reward learning across the task was then computed by subtracting the total response bias in the first block from the total response bias in the last block. Discriminability was also calculated as a measure of more general task performance ^5,7^. Response bias and discriminability were computed using the following formulae:

Response bias: $\log b=\frac{1}{2}\log\left(\frac{Rich_{correct}*Lean_{incorrect}}{Rich_{incorrect}*Lean_{correct}}\right)$

Discriminability:

Wherein a value of 0.5 was added to each cell (see ^7^ for further details). Prior to data analysis, PRT data underwent a quality-control check wherein trials with below chance accuracy and/or >10% reaction time outliers were excluded from analysis.

**2. Supplemental Results**

**List of immune markers with concentrations below the limit of detection (LOD)**

Peripheral immune analyses were conducted utilizing analytes from supernatant from LPS stimulated PBMCs with values above the limit of detection (LOD). Cytokines and chemokines with greater than 50% missing or below the LOD values were excluded from the analysis. The immune markers not included in the analysis are the following: Glial cell-line-derived neurotrophic factor (GDNF), CUB domain-containing protein 1 (CDCP1), Natural killer cell receptor 2B4 (CD244), Interleukin-7 (IL-7), osteoprotegerin (OPG), interleukin-17C (IL-17C), interleukin-17A (IL-17A), Axin-1, TNF-related apoptosis-inducing ligand (TRAIL), interleukin-20 receptor subunit alpha (IL-20RA), cystadin D (CST5), interleukin-2 receptor subunit beta (IL-2RB), interleukin-2 (IL-2), thymic stromal lymphopoietin (TSLP), stem cell factor (SCF), signaling lymphocytic activation molecule (SLAMF1), monocyte chemotactic protein 4 (MCP-4), eotaxin (CCL11), fibroblast growth factor 23 (FGF-23), interleukin-10 receptor subunit alpha (IL-10RA), fibroblast growth factor 5 (FGF-5), leukemia inhibitory factor receptor (LIF-R), C-C motif chemokine 19 (CCL19), interleukin-15 receptor subunit alpha (IL-15RA), interleukin-10 receptor subunit beta (IL-10RB), interleukin-22 receptor subunit alpha-1 (IL-22 RA1), interleukin-18 receptor-1 (IL-18R1), programmed cell death 1 ligand 1 (PD-L1), Beta-nerve growth factor (Beta-NGF), tumor necrosis factor (TNF) related activation-induced cytokine (TRANCE), interleukin-24 (IL-24), interleukin-13 (IL-13), artemin (ARTN), Fms-related tyrosine kinase 3 ligand (Flt3L), interleukin-20 (IL-20), SIR2-like protein 2 (SIRT2), interleukin-33 (IL-33), interferon gamma (IFN-gamma), fibroblast growth factor 19 (FGF-19), interleukin-4 (IL4), leukemia inhibitory factor (LIF), neurturin (NRTN), C-C motif chemokine 25 (CCL25), Fractalkine (CX3CL1), neurotrophin-3 (NT-3), interleukin-5 (IL-5), TNF-beta (TNFB), macrophage colony-stimulating factor 1 (CSF-1).

**Behavioral measures of reward (PRT)**

Fifty-one subjects completed the PRT on the assessment day. Of these, 17 healthy volunteers and 23 depressed subjects had valid data following the quality check (QC). Reasons for exclusion were: too many outliers (n=8), uncompleted task (n=1), and below chance accuracy (n=2). Depressive and anhedonic symptom severity (as indexed by the total MADRS and SHAPS scores) did not differ between subjects who passed QC and those who failed, indicating that depressive and anhedonic symptom severity was unrelated to the rate of data exclusion. The remaining analysis were conducted only on the subjects who passed the QC (n=40). As hypothesized and consistent with prior reports, response bias was higher in healthy compared to depressed [t_38_=2.27 p=.029)]. See **Figure S4**. There was no Group × Block [F(2,76) p=.89, η²=.003] interaction or main effect of Group [F(1,34) p=.50, η²=.013] for discriminability, indicating that response bias findings were not due group differences in task difficulty.

**3. Supplemental Tables and Figures**

**Table S1. Plasma level (NPX) of inflammatory factors in depressed (n=30) and healthy (n=21) subjects.**

|  | MDD | | HC | | *p value* |
| --- | --- | --- | --- | --- | --- |
|  | *M* | *SD* | *M* | *SD* |  |
| IL-15RA | 0.2507417 | 1.69259193 | 0.27681852 | 0.47967491 | 0.9 |
| IL-10RA | 0.4691416 | 0.31293645 | 0.61460481 | 0.50026079 | 0.2 |
| NT-3 | 1.17667183 | 0.38946058 | 1.21515919 | 0.31648404 | 0.7 |
| FGF-23 | 1.70913253 | 0.68233117 | 1.64095181 | 0.29242414 | 0.7 |
| SLAMF-1 | 1.12312917 | 0.34047623 | 1.0610181 | 0.26057393 | 0.5 |
| OSM | 0.77234563 | 0.5642231 | 0.81081152 | 0.56368606 | 0.8 |
| IL10 | 1.82878957 | 0.50415387 | 1.72025671 | 0.24979401 | 0.4 |
| TGF-alpha | 1.13565903 | 0.2858431 | 1.14885652 | 0.17459237 | 0.8 |
| LIF-R | 1.3052492 | 0.30827285 | 1.3120969 | 0.23855049 | 0.9 |
| Beta-NGF | 0.9363215 | 0.33906695 | 1.21014781 | 1.06974573 | 0.2 |
| MCP-4 | 2.91922407 | 0.76200003 | 3.14780314 | 0.755411 | 0.3 |
| CD6 | 2.911839 | 0.56930617 | 3.31936338 | 1.00225596 | 0.07 |
| SCF | 8.289989 | 0.49228129 | 8.27680271 | 0.48191679 | 0.9 |
| TNFSF14 | 3.59614837 | 0.69912122 | 3.86681376 | 0.81340864 | 0.2 |
| CCL4 | 5.57672907 | 1.13076778 | 5.49475138 | 0.82093693 | 0.7 |
| IL18 | 6.37292943 | 0.505215 | 6.64590895 | 0.64509475 | 0.1 |
| CCL11 | 6.4736086 | 0.51307325 | 6.57811095 | 0.4354037 | 0.4 |
| CSF-1 | 6.1417463 | 0.33266261 | 6.14798805 | 0.32132928 | 0.9 |
| CXCL1 | 8.8360257 | 1.03428263 | 9.10628538 | 0.72589558 | 0.3 |
| CST5 | 4.79474463 | 0.65450295 | 4.899812 | 0.67596726 | 0.6 |
| TRAIL | 6.33968923 | 0.37007337 | 6.4899831 | 0.25725292 | 0.1 |
| AXIN1 | 5.5378647 | 0.86713947 | 5.8364329 | 0.97780653 | 0.3 |
| CXCL11 | 7.85385637 | 1.03418542 | 8.04086895 | 1.01256817 | 0.5 |
| MCP-1 | 8.7746976 | 0.47449352 | 8.78345995 | 0.33528592 | 0.9 |
| IL-6 | 2.26955243 | 0.68891266 | 2.04075933 | 0.59192687 | 0.2 |
| uPA | 8.9855602 | 0.36214534 | 9.11451729 | 0.28262622 | 0.2 |
| LAP TGF-beta 1 | 6.46892183 | 0.64690814 | 6.73048771 | 0.7032446 | 0.2 |
| OPG | 8.68955457 | 0.33286838 | 8.79399481 | 0.44258552 | 0.3 |
| IL7 | 3.4762895 | 0.78702951 | 3.73106781 | 0.75948874 | 0.2 |
| CD244 | 4.9348823 | 0.47210952 | 5.1568609 | 0.83578879 | 0.2 |
| CDCP1 | 1.43872033 | 0.51946709 | 1.79023138 | 0.90914406 | 0.09 |
| VEGFA | 8.17609383 | 0.53510439 | 8.28244776 | 0.70172477 | 0.5 |
| CXCL9 | 5.3385674 | 0.65021925 | 5.34171986 | 0.50248081 | 0.9 |
| IL-18R1 | 5.19602017 | 0.42390135 | 5.4335541 | 0.4005575 | 0.05 |
| MMP-1 | 12.3188145 | 1.1227931 | 12.4132319 | 0.96249635 | 0.8 |
| CCL25 | 3.84674397 | 0.59909238 | 3.90272481 | 0.61720543 | 0.7 |
| DNER | 6.41080977 | 0.2679902 | 6.57354476 | 0.34281856 | 0.06 |
| EN-RAGE | 1.5829904 | 0.64479794 | 1.74495986 | 0.46543862 | 0.3 |
| CD40 | 9.58100207 | 0.60860767 | 9.9113889 | 0.84216271 | 0.1 |
| FGF-19 | 6.21027397 | 0.91903501 | 6.38831481 | 1.06777988 | 0.5 |
| MCP-2 | 7.55533017 | 0.97872691 | 7.54126929 | 0.94019578 | 0.9 |
| CASP-8 | 3.10134517 | 0.95382005 | 3.06914062 | 0.55177866 | 0.9 |
| CX3CL1 | 3.99256657 | 0.52146287 | 4.04636676 | 0.38264621 | 0.7 |
| SIRT2 | 4.79286207 | 1.06219577 | 4.95851662 | 1.05566647 | 0.6 |
| TNFRSF9 | 4.24342187 | 0.3216268 | 4.22504876 | 0.33052581 | 0.8 |
| TWEAK | 7.9997142 | 0.365616 | 8.13607157 | 0.43948932 | 0.2 |
| CCL20 | 4.32900983 | 0.80767624 | 4.15842638 | 0.60449324 | 0.4 |
| ST1A1 | 4.19342803 | 0.82420016 | 4.15965805 | 0.6617979 | 0.9 |
| STAMPB | 5.73710177 | 0.96309742 | 6.06959652 | 0.99913911 | 0.2 |
| ADA | 3.07476443 | 0.79156852 | 3.183806 | 0.57165405 | 0.6 |
| CCL28 | 1.2147415 | 0.52307566 | 1.30043443 | 0.45050076 | 0.5 |
| 4E-BP1 | 7.28459853 | 0.9905263 | 7.50386681 | 0.97390462 | 0.4 |
| FGF-21 | 3.3670251 | 1.42110334 | 3.776784 | 1.15376615 | 0.3 |
| HGF | 6.2835528 | 0.29455723 | 6.36339881 | 0.40985437 | 0.4 |
| CCL19 | 7.59043667 | 0.94954694 | 7.2892559 | 0.63861609 | 0.2 |
| IL-10RB | 5.16839913 | 0.31077218 | 5.29500162 | 0.33033965 | 0.2 |
| TNFB | 2.42439417 | 0.29459122 | 2.36935686 | 0.4022132 | 0.6 |
| PD-L1 | 3.0591828 | 0.58578016 | 3.21521171 | 0.64774684 | 0.4 |
| CXCL5 | 10.9932025 | 1.31021758 | 11.3808214 | 0.85045702 | 0.2 |
| TRANCE | 2.91702963 | 0.74489817 | 3.20091481 | 0.52055623 | 0.1 |
| IL-12B | 2.26043253 | 0.40589906 | 2.44490252 | 0.45447655 | 0.1 |
| CXCL10 | 7.36428663 | 0.871295 | 7.28763786 | 0.45977333 | 0.7 |
| MMP-10 | 5.10274113 | 0.64888642 | 5.04890257 | 0.43018933 | 0.7 |
| CCL23 | 7.98442833 | 0.66097555 | 8.07429576 | 0.55754653 | 0.6 |
| CD5 | 3.2543668 | 0.41097315 | 3.36995786 | 0.63111772 | 0.4 |
| CCL3 | 3.64225083 | 1.10206896 | 3.7114221 | 0.79855213 | 0.8 |
| Flt3L | 7.0405771 | 0.37236102 | 7.13386752 | 0.35392723 | 0.4 |
| CXCL6 | 7.10443867 | 1.019317 | 7.59552024 | 1.07001051 | 0.1 |
| IL8 | 4.80137987 | 0.67388224 | 5.40300576 | 1.36746663 | 0.09 |

**Figure S1. Schematic representation of the Incentive Flanker Task (IFT)**. The IFT was used during the fMRI to measure brain responses to reward expectancy (or anticipation) and feedback response (or consumption).


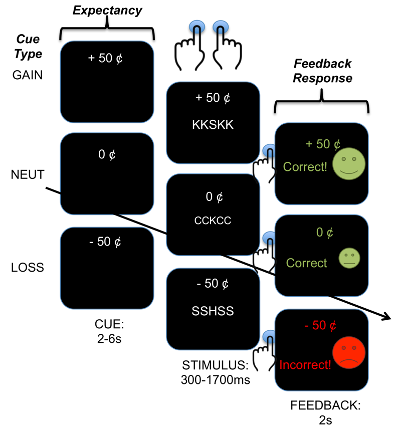


**Figure S2. Scree plot of the eigenvalues of factors or principal components analysis (PCA)**

**Figure S3. Reward Learning in Patients with Unipolar Depressive Disorder (n=23) and Healthy Volunteers (n=17). A.** Reward learning (computed by subtracting the total response bias in the first block from the total response bias in the last block) difference between depressed subjects and healthy volunteers (t_38_=2.27, p=.029). **B.** Mean response bias across the three blocks of the Probabilistic Reward Task (PRT) in depressed subjects and healthy volunteers. *p<.05, error bars represent standard error of the mean (SEM)

**4. Supplementary References**

1 Kundu P, Inati SJ, Evans JW, Luh W-M, Bandettini PA. Differentiating BOLD and non-BOLD signals in fMRI time series using multi-echo EPI. *Neuroimage* 2012; **60**: 1759–1770.

2 Kundu P, Brenowitz ND, Voon V, Worbe Y, Vértes PE, Inati SJ *et al.* Integrated strategy for improving functional connectivity mapping using multiecho fMRI. *Proc Natl Acad Sci USA* 2013; **110**: 16187–16192.

3 Kundu P, Voon V, Balchandani P, Lombardo MV, Poser BA, Bandettini PA. Multi-echo fMRI: A review of applications in fMRI denoising and analysis of BOLD signals. *Neuroimage* 2017; **154**: 59–80.

4 Stern ER, Welsh RC, Fitzgerald KD, Gehring WJ, Lister JJ, Himle JA *et al.* Hyperactive error responses and altered connectivity in ventromedial and frontoinsular cortices in obsessive-compulsive disorder. *Biol Psychiatry* 2011; **69**: 583–591.

5 Pizzagalli DA, Jahn AL, O’Shea JP. Toward an objective characterization of an anhedonic phenotype: a signal-detection approach. *Biol Psychiatry* 2005; **57**: 319–327.

6 Vrieze E, Pizzagalli DA, Demyttenaere K, Hompes T, Sienaert P, de Boer P *et al.* Reduced reward learning predicts outcome in major depressive disorder. *Biol Psychiatry* 2013; **73**: 639–645.

7 Pizzagalli DA, Iosifescu D, Hallett LA, Ratner KG, Fava M. Reduced hedonic capacity in major depressive disorder: evidence from a probabilistic reward task. *J Psychiatr Res* 2008; **43**: 76–87.
